# Transcriptome Identified lncRNAs Associated with Renal Fibrosis in UUO Rat Model

**DOI:** 10.3389/fphys.2017.00658

**Published:** 2017-08-31

**Authors:** Jiazeng Sun, Shang Zhang, Bianhua Shi, Dexian Zheng, Juan Shi

**Affiliations:** National Laboratory of Medical Molecular Biology, Institute of Basic Medical Sciences, Chinese Academy of Medical Sciences and Peking Union Medical College Beijing, China

**Keywords:** lncRNA, renal fibrosis, UUO, TGF-β, urine

## Abstract

Renal fibrosis represents a final common outcome of many renal diseases and has attracted a great deal of attention. To better understand whether lncRNAs could be a player in this process or be a biomarker for renal fibrosis diagnosis, we compared transcriptome sequencing data on renal tissues and urine respectively between UUO (unilateral ureteral obstruction) and shamed (Sham) rat model. Numerous genes including lncRNAs with significant changes in their expression were identified. 24 lncRNAs were up-regulated and 79 lncRNAs were down-regulated in the renal tissues of the UUO rats. 625 lncRNAs were up-regulated and 177 lncRNAs were down-regulated in urines of the UUO rats. Among the lncRNAs upregulated in renal tissue of UUO rats, 19 lncRNAs were predicted containing several conserved Smad3 binding motifs in the promoter. Among them, lncRNAs with putative promoter containing more than 4 conserved Smad3 binding motifs were demonstrated to be induced by TGF-β significantly in normal rat renal tubular epithelial NRK-52E cells. We further confirmed that lncRNA TCONS_00088786 and TCONS_01496394 were regulated by TGF-β stimulation and also can influence the expression of some fibrosis-related genes through a feedback loop. Based on transcriptome sequencing data, bioinformatics analysis and qRT-PCR detection, we also demonstrated lncRNA in urine are detectable and might be a novel biomarker of renal fibrosis. These results provide new information for the involvement of lncRNAs in renal fibrosis, indicating that they may serve as candidate biomarkers or therapeutic targets in the future.

## Introduction

Chronic kidney disease (CKD) is a major problem for personal health and social development, especially for the aging population (Nitta et al., 2013; Mallappallil et al., 2014; Tonelli and Riella, 2014). According the 2010 Global Burden of Disease study, in the list of causes of total number of deaths worldwide, CKD climbed the list from 27th to 18th in 1990 and 2010 (Jha et al., 2013). Renal biopsies are commonly used to assess the degree of tubulointerstitial fibrosis. However, renal biopsy is invasive and potentially associated with severe side effects, and making repeated monitoring become practically difficult. Hence, reliable noninvasive biomarkers for monitoring renal disease are urgently needed in the clinical management of patients with CKD.

Renal inflammation and fibrosis are common pathological conditions of CKD (Bohle et al., 1987; Wynn, 2007). It is well established that renal fibrosis is caused by a loss of functioning nephrons along with accumulation of fibroblasts and excessive matrix proteins (Eddy and Neilson, 2006). A number of molecular modulators such as cytokines, growth factors, metabolic toxins, and stress molecules were reported regulate the progression of renal fibrosis (Gajjala et al., 2015). Among them, transforming growth factor-β (TGF-β) plays a key role in the pathogenesis of renal fibrosis (Bottinger, 2007; Meng et al., 2016). In the process, TGF-β bind to the specific receptor II, therefore activate the TGF-β receptor type I (TβRI)-kinase, causing phosphorylation of Smad2 and Smad3. And then, phosphorylated Smad2 and Smad3 recruit Smad4 forming the Smad complex, being a transactivator, which translocates into the nucleus to regulate the renal fibrosis related target gene transcription (Derynck and Zhang, 2003; Wrana, 2013).

Long noncoding RNAs (lncRNAs) with the length of more than 200 nt, are a large class of non-protein-coding transcripts, which play significant roles in numerous physiological and pathological processes (Batista and Chang, 2013; Melissari and Grote, 2016; Schmitt and Chang, 2016). Although increasing lncRNAs are identified, a large number of them have not been functionally characterized.

Urine is a potentially better biological source of biomarkers for monitoring kidney dysfunction, because this fluid is the direct excreta of the kidney (Li et al., 2014). Urine-based diagnostic tests have the advantage of being non-invasive or minimally invasive. Yuan et al. (2015) identified urinary biomarkers related to renal tubular injury and interstitial fibrosis in rat UUO model using urinary proteomic profiling. And they found significantly increased levels of Vimentin, Annexin A1, and Clusterin might serve as candidate biomarkers of interstitial fibrosis. Compared with protein biomarkers, RNA biomarkers showed more sensitivity and specificity. The simplicity of the qRT-PCR makes them accessible for routine use. PCR enables traces of RNA sequences to be amplified and thus captured specifically with high sensitivity. Moreover, the cost of RNA biomarker is much lower than protein biomarker because detecting each protein requires a specific antibody. Currently, PCA3 is the most extensively studied long noncoding RNA as urine biomarker. PCA3 is a prostate-specific non-coding RNA that is highly overexpressed in prostate cancer compared to the normal prostate (Bussemakers et al., 1999; Hessels et al., 2004). The US Food and Drug Administration has approved PCA3 RNA-based urine test for the diagnosis of prostate cancer.

One research group performed RNA-seq analysis in the UUO mouse model and identified differentially expressed lncRNA during progression of renal fibrosis (Arvaniti et al., 2016). However, whether these lncRNAs are released into body fluids and can serve as non-invasive biomarkers remains unclear. To better understand whether lncRNAs play roles in the process of renal fibrosis and have a potential to be noninvasive biomarker for diagnosis, the full transcriptome of renal tissue and urine were identified using the RNA-seq methodology in UUO rat renal fibrosis model. Useful information regarding modulated genes and biological processes were enriched, and randomed selected differentially expressed lncRNAs were verified by qRT-PCR assay. A few lncRNAs were predicted to be regulated through TGF-β pathway. We further confirmed that lncRNA TCONS_00088786 and TCONS_01496394 were regulated by TGF-β stimulation and also can influence the expression of some fibrosis-related genes through a feedback loop, which gave us a clue to further investigate the function and mechanism of the specific selected lncRNAs and develop a novel diagnostic method for renal fibrosis.

## Materials and methods

### Construction of shamed and UUO rat model

The experiments use all rats with 8 weeks of age. The rats were divided into two groups, including one undergoing ligation of the right ureter as UUO rat model, the other just under the same surgery but not ligation the ureters as shamed rat model. After 2 weeks, we collected the kidneys and stored in liquid nitrogen for further analysis. All the animal experiments were performed in accordance with the institutional guidelines for animal care and approved by the Institutional Animal Care and Use Committee of the Chinese Academy of Medical Sciences.

To estimate the grade of interstitial fibrosis, the interstitial area that was stained green with Masson's trichrome was evaluated as a percentage of the total examined area in five randomly chosen sections prepared from each kidney sample. For each section, interstitial space widening with focal leukocyte infiltration and interstitial fibrosis was assessed in high-power fields to quantify the results. The Banff classification of kidney pathology was used for scoring the degree of mononuclear cell infiltration and interstitial fibrosis. The score was graded from 0 to 3, depending on the severity of histological characteristics (Ozbek et al., 2009). All the kidney tissues from UUO rats used in the study were grades 2 or 3.

Urine samples were collected based on previous protocol (Yuan et al., 2015). Urine was collected from the residuary ureter linked to the kidney by ureter catheterization for 3 h before the rats were sacrificed. In first 0.5 h, the urine collected had accumulated during induction of UUO, and in the final 2.5 h, newly generated urine from the kidney injured by UUO was collected. Only the urine collected during the final 2.5 h was used in the following experiments. Urine from the sham-operated rats was collected from the ureter in the same manner as for the UUO rats. The kidney was then harvested. One half of the kidney was fixed, and the other half was used for RNA extraction.

### RNA isolation, library preparation and sequencing

RNA was isolated from kidney tissues from two groups in three duplications, respectively. RNA qualification was monitored on 1% agarose gels. RNA purity was checked using the NanoPhotometer® spectrophotometer (IMPLEN, CA, USA). RNA concentration was measured using Qubit® RNA Assay Kit in Qubit® 2.0 Flurometer (Life Technologies, CA, USA). RNA integrity was assessed using the RNA Nano 6000 Assay Kit of the Bioanalyzer 2100 system (Agilent Technologies, CA, USA). A total amount of 3 μg RNA per sample was used as input material for the RNA sample preparations. After removal of ribosomal RNA by Epicentre Ribo-zeroTM rRNA Removal Kit (Epicentre, USA), the residual RNA was used for constructing sequencing libraries by using the rRNA-depleted RNA by NEBNext® UltraTM Directional RNA Library Prep Kit for Illumina® (NEB, USA) following manufacturer's recommendations. First strand cDNA was synthesized using random primer and second strand cDNA synthesis was subsequently performed. In the reaction buffer, dNTPs with dTTP were replaced by dUTP. Remaining overhangs were converted into blunt ends via exonuclease/polymerase activities. After adenylation of 3′ ends of DNA fragments, NEBNext Adaptor with hairpin loop structure were ligated to prepare for hybridization. In order to select cDNA fragments of preferentially 150 ~ 200 bp in length, the library fragments were purified with AMPure XP system (Beckman Coulter, Beverly, USA). Then 3 μl USER Enzyme (NEB, USA) was used with size-selected, adaptor-ligated cDNA at 37°C for 15 min followed by 5 min at 95°C before PCR. Then PCR was performed with Phusion High-Fidelity DNA polymerase, Universal PCR primers and Index (X) Primer. At last, products were purified (AMPure XP system) and library quality was assessed on the Agilent Bioanalyzer 2100 system.

### Bioinformatics analysis

The sequencing data is publicly upload to the Gene Expression Omnibus database (https://www.ncbi.nlm.nih.gov/geo/query/acc.cgi?acc=GSE100633). After sequencing, clean reads were obtained by removing reads containing adapter, reads containing ploy-N and low quality reads from raw data. At the same time, Q20, Q30 and GC content of the clean data were calculated. All the downstream analyses were based on the clean data with high quality. All the clean reads were mapped to the reference genome. Index of the reference genome was built using Bowtie v2.0.6 and paired-end clean reads were aligned to the reference genome using TopHat v2.0.9. The mapped reads of each sample were assembled by both Scripture (beta2) and Cufflinks (v2.1.1) in a reference-based approach. Transcripts predicted with coding potential by either or all of the four tools [CNCI (Coding-Non-Coding-Index) (v2), CPC (Coding Potential Calculator) (0.9-r2), Pfam Scan (v1.3) and PhyloCSF (phylogenetic codon substitution frequency) (v20121028)] were filtered out, and those without coding potential were our candidate set of lncRNAs. GO analysis of differentially expressed genes was implemented using DAVID in which gene length bias was corrected. GO terms with corrected *P*-value less than 0.05 were considered significantly enriched by differential expressed genes. KEGG is a database resource for understanding high-level functions and utilities of the biological system, such as the cell, the organism and the ecosystem, from molecular-level information, especially large-scale molecular datasets generated by genome sequencing and other high-throughput experimental technologies (http://www.genome.jp/kegg/). We used DAVID to test the statistical enrichment of differential expression genes or lncRNA target genes in KEGG pathways.

### RNA isolation from urine samples

Urine samples were centrifuged before storage at −80°C and immediately after thawing and before RNA isolation. Total RNA, including small RNAs, was isolated from 500 uL urine with the miRNeasy Mini Kit (Qiagen) according to the manufacturer's instructions. The samples were supplemented with 5 fmol Caenorhabditis elegans miRNA 39 (cel-miR-39) as an internal spike-in control.

### Quantitative real time PCR (qRT-PCR)

To validate the RNA sequencing results, qRT-PCR was performed with the StepOnePlus^TM^ Real-Time PCR Detection System (ABI). Total RNA of each sample was isolated using Trizol Reagent (Invitrogen, USA) according to the manufacturer's instructions. The first-strand cDNA used for qPCR was synthesized from the total RNA by M-MLV (Promega). The primers designed by using primer premier 5.0 and synthesized in Sangon Biotech (Shanghai) (Supplementary Table 1). The PCR reaction was set as follows: 95°C for 30 s; 40 cycles of 95°C for 20 s, 60°C for 30 s and melting curve analysis. All measurements were conducted in triplicates. The relative fold changes of gene expression were calculated according to the 2^−ΔΔCT^ method using GAPDH or 18S ribosome RNA as an internal control.

### Statistical analysis

GraphPad Prism (v6.07) was used for statistical analysis. Results of quantitative data in this study are expressed as the mean ± SD. Significant differences between groups were compared using two-tailed ANOVA via *t*-test, and differences with *P* < 0.05 were considered statistically significant for all experiments.

## Results

### Physiognomic and histopathological evaluation of UUO rats

**Figure 1:**
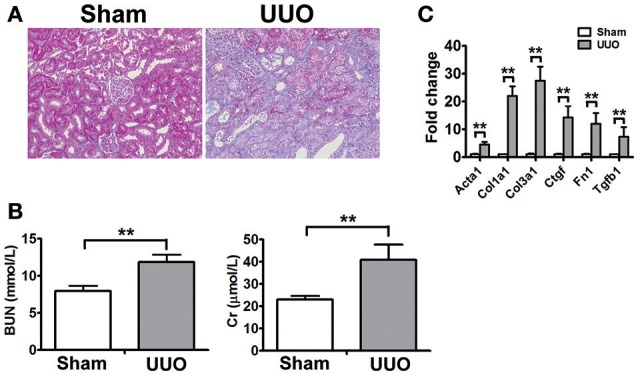
Physiognomic and histopathological evaluation of UUO rats **(A)** Masson's trichrome staining of SD rat kidneys at 2 weeks after UUO. **(B)** Blood urea nitrogen (BUN) and serum creatinine (Cr) detected in SD rat after UUO. The data represent the mean of 4 rats. **(C)** Verification of the mRNA expression of genes known to be affected in renal fibrosis. The mRNA levels of each gene were normalized to GAPDH and expressed as fold of change compared to Sham rats. Acta2, Alpha smooth muscle actin; Col1a1, Collagen alpha-1 type I; Col3a1, Collagen alpha-1 type III; Ctgf, Connective tissue growth factor; Fn1, Fibronectin 1; Tgtb1, Transforming growth factor-beta. ^*^P < 0.05 and ^**^P < 0.01 (*t*-test), *n* = 3.

To identify new molecular players and biomarkers in renal fibrosis, high throughput RNA-seq was performed in the rat UUO model. The Masson's trichrome staining of kidneys obtained from Sham and UUO rats are shown in Figure 1A. Masson's trichrome staining revealed extensive renal interstitial fibrosis in the UUO group. And all of the changes in the renal tissue suggested that the UUO model was successful and that typical fibrosis appeared 2 weeks after UUO (Figure 1A). Moreover, mean levels of blood urea nitrogen, serum creatinine increased in the UUO group compared to the Sham group (Figure 1B).

### Identification of differentially expressed genes during progression of renal fibrosis

The transcriptomic analyses were used to analyze the RNA expression in renal tissue and urine from rats in the sham-operated group and the UUO 2-week group. Renal tissues and urines were collected and total RNAs of each sample were extracted and used for libraries construction. Prior to RNA-seq analysis, total RNAs from renal tissue samples were analyzed to confirm molecular changes indicative of the fibrotic signature (Figure 1C). The libraries were then sequenced by using Illumina high throughput RNA-seq. Data analysis showed that reads of each samples were ten million level and more than 80% reads were mapped to the reference genome (Supplementary Table 2). All sample raw reads showed high quality and the genome mapped reads were classified as different types of RNA. Pearson correlation analysis and FPKM distribution (Supplementary Figure 1) showed that the expression pattern of RNA samples was similar among 3 duplications in one group. In one word, high quality data ensured reliability of subsequent analysis.

**Figure 2:**
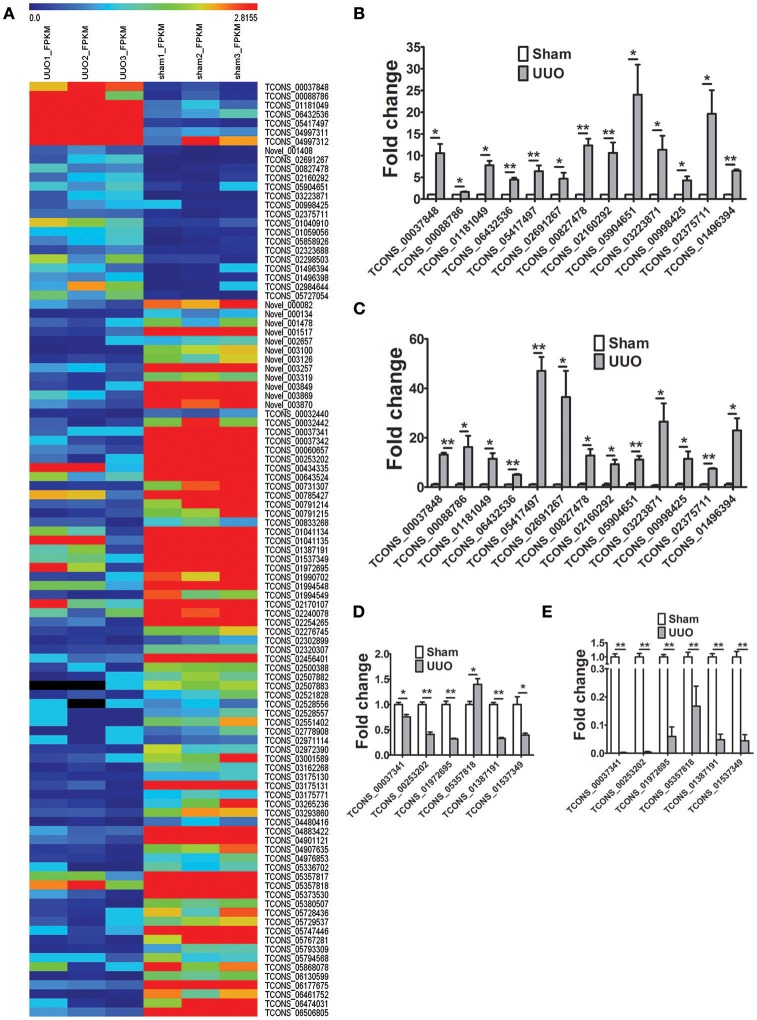
Differentially expressed genes in the UUO model of renal fibrosis. **(A)** Hierarchical clustering and heatmap showing the expression changes in the renal tissues between Sham operated and UUO rats. **(B)** Verification of the expression profiles of 13 upregulated lncRNAs in renal tissues with qRT-PCR analysis. Each sample was performed in triplicate and the gene expression levels were normalized to that of 18srRNA expression. *n* = 3–6. **(C)** Fold change expression of 13 upregulated lncRNAs in renal tissues from RNA-seq data. *n* = 3. **(D)** Verification of the expression profiles of 6 downregulated lncRNAs in renal tissues with qRT-PCR analysis. Each sample was performed in triplicate and the gene expression levels were normalized to that of 18srRNA expression. *n* = 3–6. **(E)** Fold change expression of 6 downregulated lncRNAs in renal tissues from RNA-seq data. *n* = 3. ^*^P < 0.05 and ^**^P < 0.01 (*t*-test).

The transcripts per sample defined by RPKM values (reads per kilobase of exon per million reads) were compared between the Sham and UUO rat group. An absolute fold change cutoff value of 1 in log2 scale was utilized to identify up-regulated and down-regulated genes (*p* < 0.05). To identify renal fibrosis related lncRNAs, the coding potential ability of the transcripts in all samples were predicted by using CPC, PFAM, CNCI, and phyloCSF tools. Differentially expressed genes in renal tissues between UUO and Sham group are reported in detail (Supplementary Tables 3–6). 11280 transcripts were considered as non-coding RNAs. We identified 1232 genes differentially expressed in the renal tissue of UUO rats compared with Sham group. Among then, 750 mRNAs were up-regulated and 379 mRNAs were down-regulated in the renal tissues of the UUO rats. Twenty four lncRNAs were up-regulated and 79 lncRNAs were down-regulated in the renal tissues of the UUO rats. Hierarchical clustering and heatmap (Figure 2A) showed the expression changes of lncRNA between UUO and Sham group.

Subsequently, 997 genes were differentially expressed in the urine of UUO rats compared with Sham group. Among then, 168 mRNAs were up-regulated and 27 mRNAs were down-regulated in the renal tissues of the UUO rats. Six hundred and twenty five lncRNAs were up-regulated and 177 lncRNAs were down-regulated in the renal tissues of the UUO rats. Differentially expressed genes in urine were reported in detail (Supplementary Tables 7–10).

19 randomly selected lncRNAs dysregulated in renal tissues in UUO rats were validated in qRT-PCR analysis. As shown in Figures 2B,D, the results showed that for 18 of the 19 lncRNAs, qRT-PCR revealed the same expression tendency as the RNA-Seq data (Figures 2C,E), despite some quantitative differences in expression levels.

### Analysis of cellular processes, molecular pathways and specific genes involved in renal fibrosis

**Figure 3:**
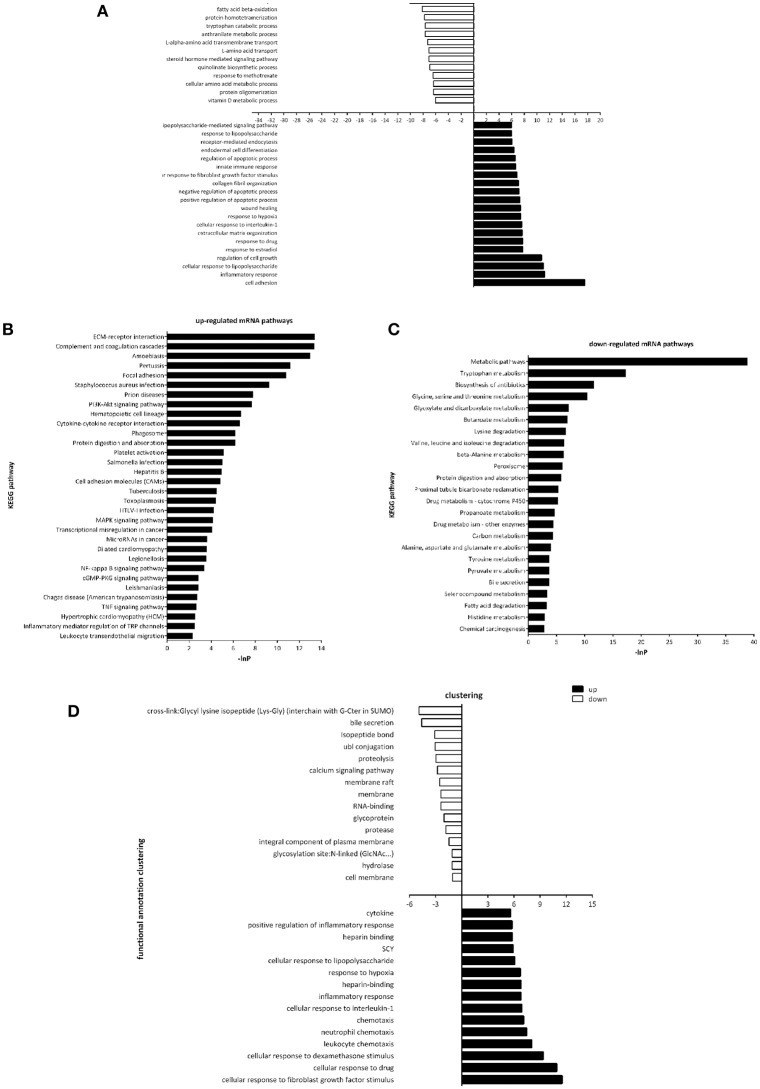
Gene Ontology and Pathway analysis of differentially expressed genes. **(A)** Gene ontology enrichment of dysregulated genes in the renal tissues between Sham operated and UUO rats. **(B)** Up-regulated mRNA pathways when comparing Sham operated vs. UUO rats. **(C)** Down-regulated mRNA pathways when comparing Sham operated vs. UUO rats. **(D)** Gene ontology enrichment of dysregulated genes in the urine between Sham operated and UUO rats.

Gene Ontology (GO) and pathway analysis of differentially expressed transcripts were performed to identify the cellular processes and molecular pathways. Of note, GO term analysis of the dysregulated genes was enriched in terms related to cell adhesion, inflammatory response, regulation of cell growth, regulation of apoptotic process, would healing, etc. (Figure 3A), which were already reported to be associated with fibrosis. Next, by pathway analysis we documented a number of biological pathways characterized as up- or down-regulated (Figures 3B,C). GO term analysis of the dysregulated genes in the urine of UUO rats was enriched in terms related to cellular response to fibroblast growth factor stimulus, inflammatory response, chemotaxis, etc. (Figure 3D), which indicated that differentially expressed transcripts in urine reflected the real progress of renal fibrosis.

### IncRNAs might affect renal fibrosis through TGF-β pathway

Many lncRNAs may function in cis to regulate expression of nearby genes. Therefore, we performed further bioinformatics searches 100 kb upstream and 100 kb downstream of all the dysregulated lncRNA genes for the identification of protein-coding genes. The examination of the lncRNA's genomic loci revealed a large number of protein-coding genes that could be potentially affected in cis by the differential expression of the corresponding lncRNAs (listed in Supplementary Table 11). And some of these proteins like CTSD, ATF3, WNT5A were reported play important roles in renal fibrosis or other renal disease.

**Figure 4:**
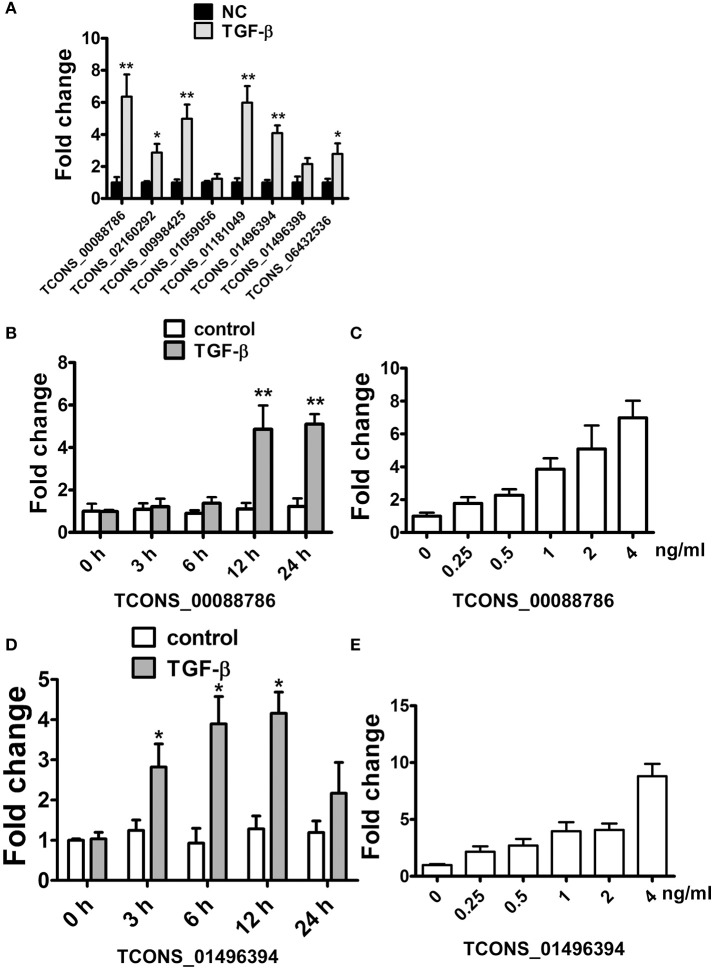
TGF-β induced lncRNA expression in NRK52E cells. **(A)** qRT-PCR showed that 12 hTGF-β (2 ng/ml) treatment significantly induced several lncRNAs upregulation in NRK-52E cells. **(B)** qRT-PCR showed that TGF-β induced TCONS_00088786 expression in a time-dependant manner. **(C)** qRT-PCR showed that TGF-β induced TCONS_00088786 expression in a dose-dependant manner. **(D)** qRT-PCR showed that TGF-β induced TCONS_01496394 expression in a time-dependant manner. **(E)** qRT-PCR showed that TGF-β induced TCONS_01496394 expression in a dose-dependant manner. ^*^P < 0.05 and ^**^P < 0.01 (*t*-test), *n* = 3.

As Transforming growth factor-β (TGF-β), a fibrosis stimulator, could induce intracellular Smad protein active to be a transcription factor thus play a key role in the pathogenesis of renal fibrosis, each putative promoter (5 kb upstream of transcription start) of up-regulated lncRNAs were analyzed to identify the potential binding site (CAGACA) of Smad3. As listed in Supplementary Table 12, 19 lncRNAs were predicted containing several conserved Smad3 binding motifs. We further studied whether these lncRNAs were regulated by TGF-β in rat proximal tubular epithelial NRK-52E cells. LncRNAs with putative promoter containing more than 4 conserved Smad3 binding motifs were selected. As shown in Figure 4A, TGF-β1 significantly induced these several lncRNAs upregulation in NRK-52E cells which indicated that these lncRNA involved in TGF-β pathway.

**Figure 5:**
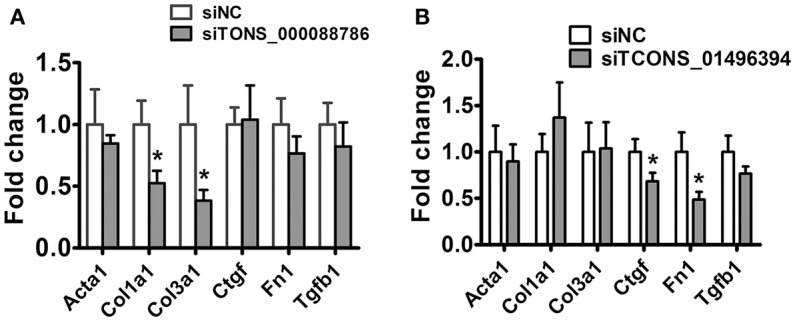
lncRNAs might affect renal fibrosis through TGF-β pathway. **(A,B)** mRNA expression profile of genes related to fibrosis in TCONS_00088786 knockdown and TCONS_01496394 knockdown, as indicated. The mRNA levels of all genes of interest were normalized to GAPDH and expressed as fold of change to control. ^*^P < 0.05 and ^**^P < 0.01 (*t*-test), *n* = 3.

Following this analysis, and based on proximity of lncRNAs to protein-coding genes with important functions in renal fibrosis as well as on the fold change in their expression levels, we focused on TCONS_00088786 and TCONS_01496394 for further study. The relationship between TGF-β/Smad signaling and TCONS_00088786, TCONS_01496394 expression was delineated in the NRK52E cells *in vitro*. As shown in Figures 4B–E, addition of TGF-β was able to induce TCONS_00088786 and TCONS_01496394 expression in a time- and dosage-dependent manner. The putative function of TCONS_00088786 and TCONS_01496394 in TGF-β-induced fibrosis was determined *in vitro* by transiently transfecting the siRNA (siRNA sequence was listed in Supplementary Table 13) into NRK52E cells then detecting a panel of fibrosis-related genes. The knockdown of the lncRNAs was evaluated by qRT-qPCR (Supplemental Figure 2). As shown in Figures 5A,B, lncRNAs affects the expression of fibrosis-related genes in a different fashion: TGF-β–induced expression of Col1a1 and Col3a1 collagen genes was significantly inhibited by TCONS_00088786 siRNA and TGF-β-induced expression of Ctgf and Fn1 genes was significantly inhibited by TCONS_01496394 siRNA. The results suggested that TCONS_00088786 and TCONS_01496394 might be critically involved in renal fibrosis, with being regulated by TGF-β/Smad signaling, forming a feedback loop to regulate renal fibrosis.

### IncRNA in urine are detectable and might be a novel biomarker

**Figure 6:**
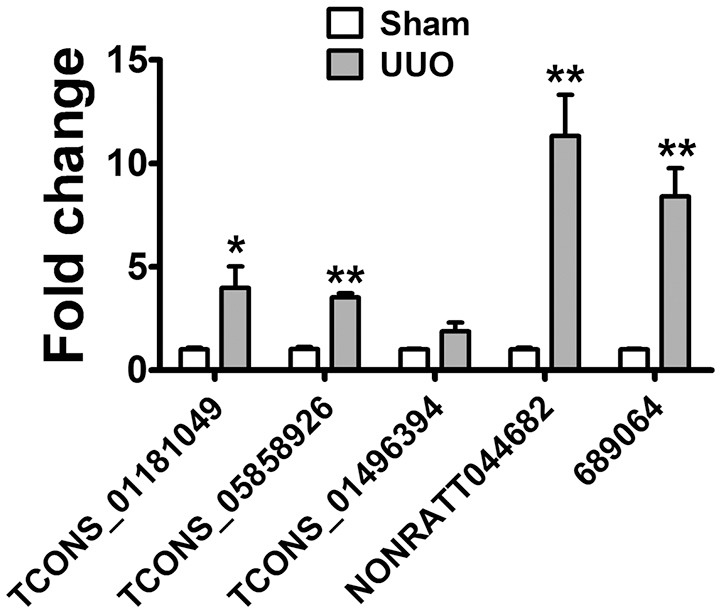
lncRNA in Urine are detectable and might be a novel biomarker. Verification of the expression profiles of lncRNAs in urine of UUO rats with qRT-PCR analysis. Each sample was performed in triplicate and the gene expression levels were normalized to that of cel-miR-39 expression. ^*^P < 0.05 and ^**^P < 0.01 (*t*-test), *n* = 3.

The detection of lncRNAs in urine is challenging owing to their low abundance. The RNA-seq results showed that lncRNAs in urine were detectable with enough diversity and abundance (Supplementary Tables 9, 10). Some lncRNAs including NONRATT044682, 361619, 689064 showed great abundance in the urine of UUO rats suggesting the possibility of urinary lncRNAs as candidate biomarker of renal fibrosis. Meanwhile, 7 dysregulated lncRNAs (5 upregulted and 2 downregulated) showed same variation tendency in renal tissues and urine of UUO rats (Supplementary Table 14). And 5 upregulated lncRNAs were predicted containing several conserved Smad3 binding motifs, further demonstrating the reliability of these lncRNAs to be a potential target of renal fibrosis. The expression of these upregulated lncRNAs (TCONS_01181049, TCONS_05858926, TCONS_02298503, TCONS_01496394, TCONS_01496398, NONRATT044682, 361619, 689064) in urine was validated in qRT-PCR analysis. As shown in Figure 6, TCONS_01181049, TCONS_05858926, TCONS_01496394, NONRATT044682, 689064 could be detected in the urine of UUO rats. And qRT-PCR revealed the same expression tendency as the RNA-Seq data, despite some quantitative differences in expression levels. We further analyzed the conservation of these lncRNAs to investigate the possibility of these lncRNAs to be a biomarker in the patients. As shown in Supplemental Figure 3, NONRATT044682, 689064 and TCONS_05858926 showed high conservation indicating the potential of these lncRNAs as a biomarker in the patients.

## Discussion

This study identified the characteristics of transcripts especially lncRNAs being likely to play roles in renal fibrosis process by utilized next generation sequencing. Although RNA high throughput sequencing techniques have been employed in many researches including nephrology, the underlying mechanism of renal fibrosis have not been clearly clarified. Therefore, to further address this issue, we systematically analyzed the whole transcriptome between UUO rat models of renal fibrosis and the shamed ones.

We performed whole transcriptome analysis combined with bioinformatics analysis in the UUO rat model of renal fibrosis. Of note, GO term analysis of the dysregulated genes was enriched in terms related to cell adhesion, inflammatory response, regulation of cell growth, regulation of apoptotic process, would healing, etc. These genes were already reported to be associated with fibrosis (Speca et al., 2012; Wi and Chen, 2014; Nakagawa et al., 2015). This result was also consistent with the RNA-seq data performed in the UUO mouse model (Arvaniti et al., 2016). Moreover, GO term analysis of the dysregulated genes in the urine of UUO rats was also enriched in terms related to cellular response to fibroblast growth factor stimulus, inflammatory response, chemotaxis, etc. The RNA-seq data in renal tissue and urine demonstrated that molecular changes in urine could reflect the histopathological progression of renal fibrosis.

Several lncRNAs were reported play role in renal fibrosis (Zhou et al., 2015; Wang et al., 2016; Yi et al., 2017). Analysis of our data led to the association of a list of lncRNAs with UUO, not previously associated with renal fibrosis. Many lncRNAs may function in cis to regulate expression of nearby genes. The examination of 100 kb upstream and 100 kb downstream of the dysregulated lncRNAs' genomic loci revealed a large number of protein-coding genes that might be potentially affected in cis by the differential expression of the corresponding lncRNAs. And some of these proteins like CTSD, ATF3, Wnt5a were reported play important roles in renal fibrosis or other kidney disease. For example, CtsD was upregulated in damaged tubular cells in nephrotoxic and ischemia reperfusion induced acute kidney injury. CtsD inhibition led to an improvement in kidney function, a reduction in apoptosis and a decrease in tubular cell damage in kidneys (Cocchiaro et al., 2016). ATF3 was demonstrated interacted directly with histone deacetylase 1 (HDAC1) and recruited HDAC1 into the ATF/NF-κB sites in the IL-6 and IL-12b gene promoters to protect against acute kidney injury (Li et al., 2010). And Wnt upregulation were detected after chronic renal injury leads to accumulation of β-catenin and induces the expression of its downstream target genes. Blockade of Wnt signaling inhibits interstitial matrix gene expression and attenuates collagen deposition and tissue scarring (He et al., 2009). These finding indicated that the lncRNAs identified in our study might play important role in renal fibrosis. Furthermore, out results suggested that TCONS_00088786 (neighbor of CtsD) and TCONS_01496394 (neighbor of ATF3) may be critically involved in renal fibrosis, with being regulated by TGF-β/Smad signaling, forming a feedback loop to regulate renal fibrosis. Therefore, our data provide new information about the involvement of lncRNAs in renal fibrosis, indicating that they may serve as candidate biomarkers or therapeutic targets of renal fibrosis.

It was reported that circulating long noncoding RNA TapSaki is a predictor of mortality in critically ill patients with acute kidney injury (Lorenzen et al., 2015). Compared to serum, urine is easily accessible and an excellent source of biomarkers in case of kidney injury. Therefore, the present study investigated the levels of lncRNAs in urine of UUO rats by whole-genome expression analysis. The RNA-seq results showed that lncRNAs in urine were detectable with enough diversity and abundance. It is note that lncRNAs had low sequence conservation compared to protein coding genes. It is important to perform homologous analysis of the dysregulated lncRNAs identified in UUO rat with human lncRNAs. It was shown that some lncRNAs showed high conservation indicating the potential of these lncRNAs as a biomarker in the patients.

The novel biomarkers are needed for early diagnosis and prognosis of the disease. In future studies, other animal models or models with different stages may provide better candidate early biomarkers. In addition, clinical samples should be used to verify the lncRNAs as clinically applicable biomarkers of renal fibrosis. Furthermore, studies in current literature did not clarify the mechanism of lncRNAs. Detailed studies on selected lncRNA now underway in our laboratory to further investigate the mechanisms of the lncRNA in renal ficrosis.

## Ethics statement

All of animal experiments were performed in adherence to the Animal Experimental Committee of PUMC in China.

## Author contributions

JSh contributed to the conception and design of the research. JSu, SZ, and BS performed the experiments, analyzed data. JSu wrote the manuscript. SZ analyzed the data. JSh and DZ edited and revised the manuscript.

### Conflict of interest statement

The authors declare that the research was conducted in the absence of any commercial or financial relationships that could be construed as a potential conflict of interest.
