# Supplementary material for: Transcriptome Identified lncRNAs Associated with Renal Fibrosis in UUO Rat Model
Source: Front Physiol. 2017 Aug 31;8:658. doi: 10.3389/fphys.2017.00658 (PMC5583212; doi:10.3389/fphys.2017.00658)
Supplement: Supplementary file 1 [file DataSheet1.DOC]

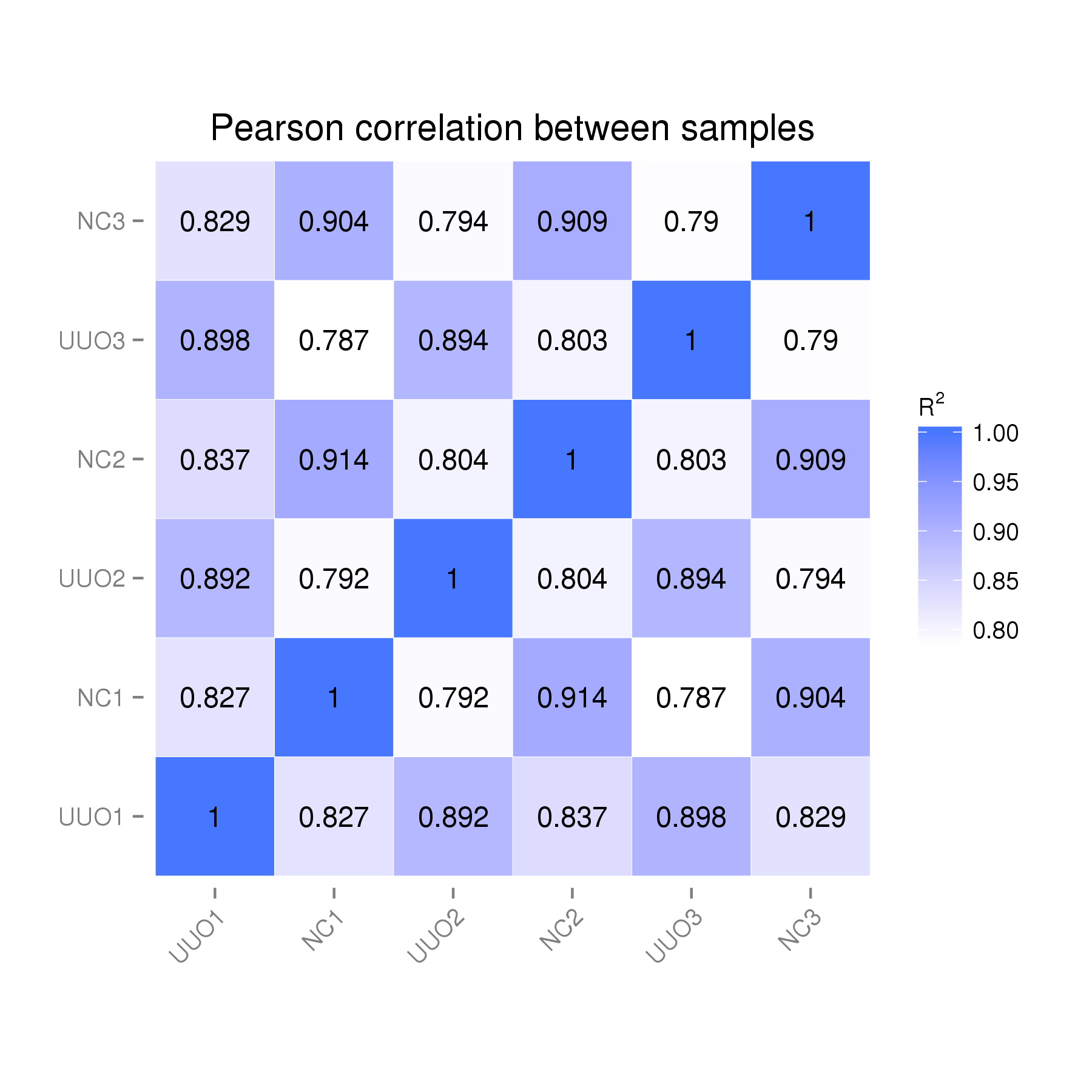


**A**

**B**

**C**


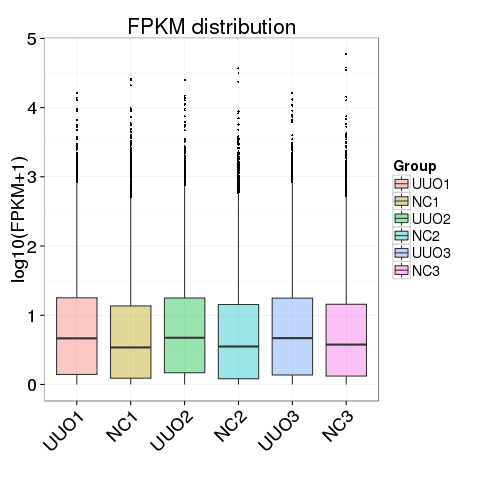


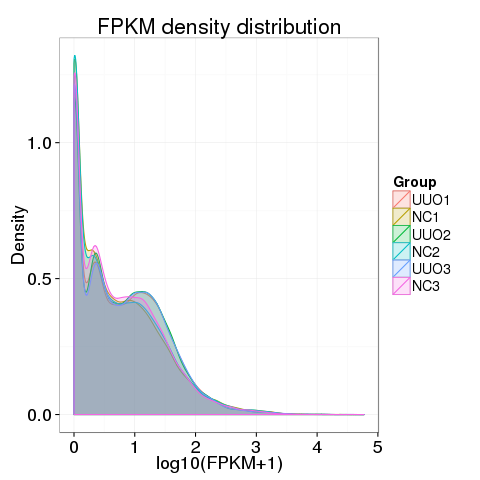


**Supplementary Figure 1**. Pearson correlation analysis (A) and FPKM distribution (B, C) showed that the expression pattern of RNA samples was similar among 3 duplications in UUO group and Sham group.

**Supplementary Figure 2**. qRT-PCR showed the expression of indicated lncRNA in the NRK-52E cells transfected with corresponding siRNA.

**689064**

**
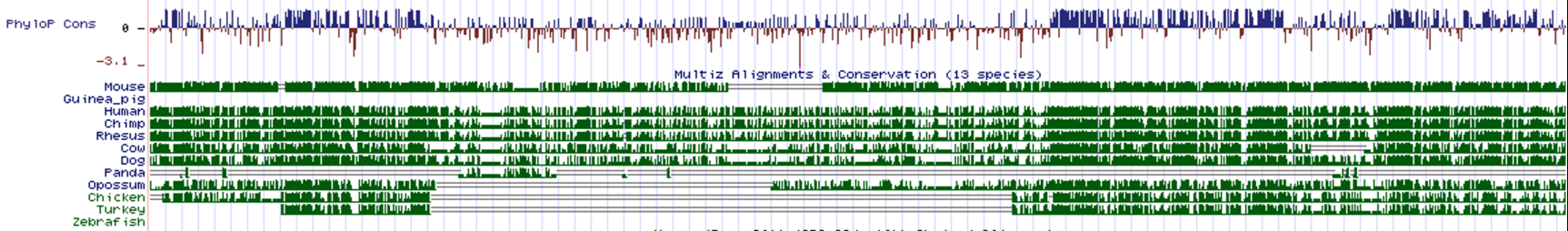
**

**NONRATT044682**

**
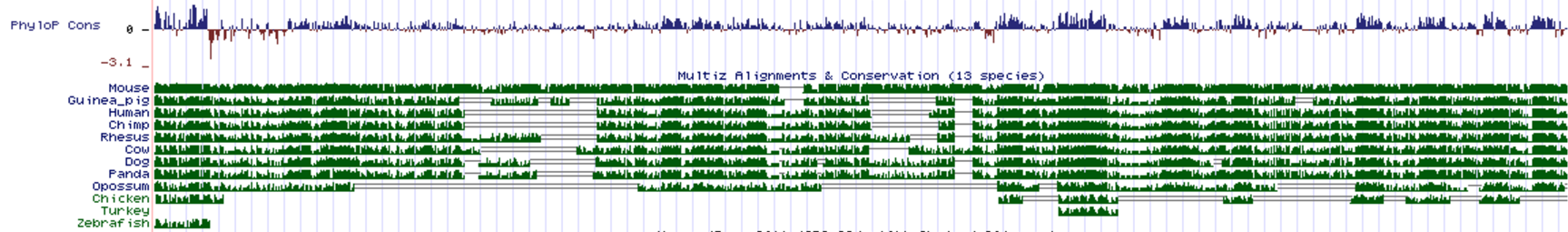
**

**TCONS_05858926**


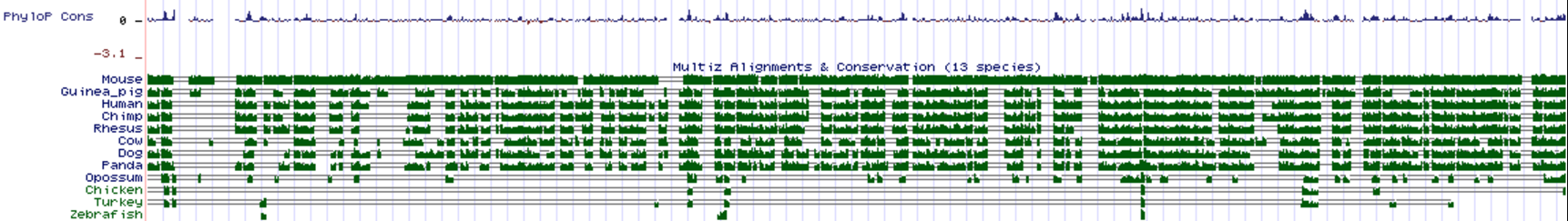


**Supplementary Figure 3.** Conservation analysis of lncRNAs in UCSC genome database.
